# Incidents of high tick load in injured cheetahs after reintroduction into a tropical ecosystem

**DOI:** 10.4102/ojvr.v92i1.2206

**Published:** 2025-04-23

**Authors:** Tamar A. Kendon, Carlos L. Pereira, Hugo Pereira, Kelsey Brown, David Gaynor, Willem D. Briers-Louw

**Affiliations:** 1 Zambeze Delta Conservation, Marromeu, Mozambique; 2 Mozambique Wildlife Alliance, Maputo, Mozambique; 3 The Metapopulation Initiative, Polokwane, South Africa; 4 Mammal Research Institute, University of Pretoria, Pretoria, South Africa; 5 Department of Conservation Ecology and Entomology, Stellenbosch University, Stellenbosch, South Africa

**Keywords:** *Acinonyx jubatus*, ectoparasites, immunocompromised, periorbital, myiasis, translocation

## Abstract

Ectoparasites can severely impact wildlife species, both through their feeding behaviour and by facilitating secondary infestations or bacterial infections. Here, we report on two cases of periorbital damage caused by ixodid tick infestations in cheetahs (*Acinonyx jubatus*), including a rare record of traumatic myiasis in wildlife. Both cheetahs were recently reintroduced to the Marromeu-Coutada Complex in Central Mozambique and had sustained relatively severe injuries. This ostensibly yielded them immunocompromised and thus more vulnerable to tick infestations, particularly when exposed to novel species. These incidents occurred during the peak hot-wet season in which adult tick abundance is likely highest. These cases provide novel insights into the risks of tick infestations for reintroduced cheetahs and the importance of intensive post-release monitoring to allow for timeous veterinary intervention.

**Contribution:**

This study falls within the scope of the journal. Ixodid ticks are of veterinary importance for wildlife and domestic animals across Africa because of the associated direct damage and potential spread of tick-borne pathogens. This study investigates two cases of tick infestations in cheetahs recently reintroduced into a tropical environment. The identified risk factors appear to be exposure to novel ectoparasites, injuries, and the hot-wet season. This highlights the need to consider ectoparasite risk when planning wildlife translocations.

## Introduction

Parasitism is a relationship between different species, where one species benefits while the host is harmed (Rózsa & Garay 2023). One example is when ectoparasites live on or burrow into the surface of a host’s skin to obtain food sources (Wall & Shearer 2008), and these harmful associations occur globally (Wall 2007). When hosts are infested by ectoparasites, their feeding can cause direct damage to skin and other subcutaneous tissues, stimulate immune responses, spread pathogens and even cause harmful behaviour (Wall 2007). The detrimental impacts on hosts can vary substantially and may only be detected in specific circumstances, such as when animals are in poor condition or parasite loads are particularly high (Wall & Shearer 2008).

Ticks are obligate, blood-feeding ectoparasites of human, wildlife and domestic animal hosts (Ostfeld et al. 2006). They are common reservoirs and vectors of pathogenic microorganisms that cause severe diseases and are considered the most important livestock pest in tropical and subtropical regions (Nasirian 2022). Ticks can considerably impact the health, reproductive success and survival of wildlife (Machtinger et al. 2023). For example, in the United States, winter tick (*Dermacentor albipictus*) infestations may cause mortalities in wild moose (*Alces alces*) (Debow et al. 2021) and captive white-tailed deer (*Odocoileus virginianus*) (Machtinger et al. 2021). Ticks have also driven mortalities in several African ungulate species by spreading tick-borne diseases, such as theileriosis and babesiosis, which were linked to animal naivety when translocated into areas where these diseases were endemic and/or the stress of capture and temporary captivity (Nijhof et al. 2005; Penzhorn 2006).

Lesions created by ticks may facilitate a secondary infestation or bacterial infection (Citino et al. 2009; Wall 2007). One of the concerns is traumatic myiasis, a largely neglected disease where dipterous larvae infest a live invertebrate host to feed on their tissue (Hall, Wall & Stevens 2016; Zumpt 1965). The larvae either form wounds after gaining access to the tissue or invade pre-existing wounds and enlarge them (Hall et al. 2016; Zumpt 1965). Traumatic myiasis can be an aural, ocular, rectal or genital condition (Hall et al. 2016). In Kenya, an outbreak of traumatic myiasis in common eland (*Taurotragus oryx*) had a negative effect on individual fitness and health, and necessitated euthanasia of severely affected individuals (Obanda et al. 2013). Records of myiasis also exist for Przewalski’s horses (*Equus ferus przewalskii*), with oestroid flies parasitising through nasal cavities and open wounds (Yan et al. 2019).

Cheetahs (*Acinonyx jubatus*) are exposed to numerous risks when translocated into new areas largely because of their extensive post-release movements, and vulnerability to predation, snaring and human–wildlife conflict (Buk et al. 2018; Weise et al. 2015). A poorly understood risk is exposure to novel ectoparasites following reintroduction. Previous records suggest that cheetahs may be infested by various ectoparasites, including fleas, ticks, mites, chiggers, lice and flies (Citino et al. 2009), with 13 tick species sampled from cheetahs in South Africa and 10 from those in Namibia (Horak et al. 2018). However, direct and potentially lethal effects of tick-feeding behaviour on cheetahs remain understudied. Here, we present two case studies of recently reintroduced cheetahs that developed tick infestations after sustaining potentially immunocompromising injuries in the Marromeu-Coutada Complex (MCC) of Central Mozambique.

## Background

An initial group of 11 cheetahs were reintroduced into the MCC (9754 km^2^) in August 2021. The MCC consists of a mosaic of vegetation communities, including seasonally flooded grassland, papyrus swamp, palm savanna and evergreen forest (Beilfuss 2016). Mean annual rainfall is 1150 mm, which is largely concentrated in the hot, summer months from December to March (Beilfuss 2016). Cheetahs were reintroduced to the MCC since historical records confirmed the species’ previous occurrence (Maugham 1914), anti-poaching efforts have greatly reduced bushmeat poaching (Briers-Louw et al. 2024), and the landscape supports abundant ungulate populations (Beilfuss 2016).

## Case descriptions

### Case 1

**FIGURE 1:**
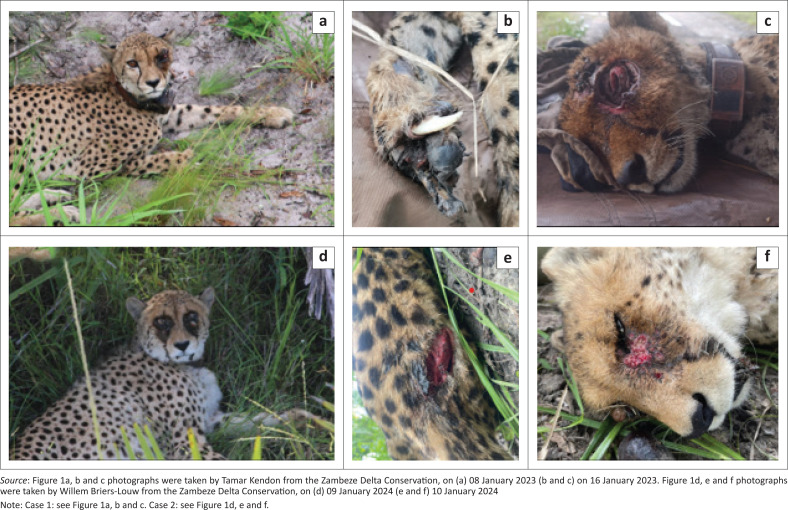
The (a and d) tick infestations, (b and e) original injuries, and (c and f) subsequent periorbital damage seen in each case study.

An adult male cheetah (~2.5 years) originating from Tswalu Kalahari Reserve, South Africa, was released into the MCC in September 2022. Less than 4 months post-release, the male presumably walked into a steel gin trap, from which he escaped, but sustained severe tissue damage to his right front foot, leaving the metacarpals exposed and the animal likely immunocompromised (Figure 1). A dysfunctional collar and the individual’s movement through dense forest at the time restricted immediate identification of the injury. During this period, the individual developed an intense infestation of ticks around the left eye and genitoanal area. Following immobilisation for veterinary treatment, it was discovered that traumatic myiasis had severely damaged his left eye and fly larvae were visible in the ocular wound. Owing to the severity, surgical repair was not possible, and the individual was euthanised. No ticks were collected from this individual.

### Case 2

**FIGURE 2:**
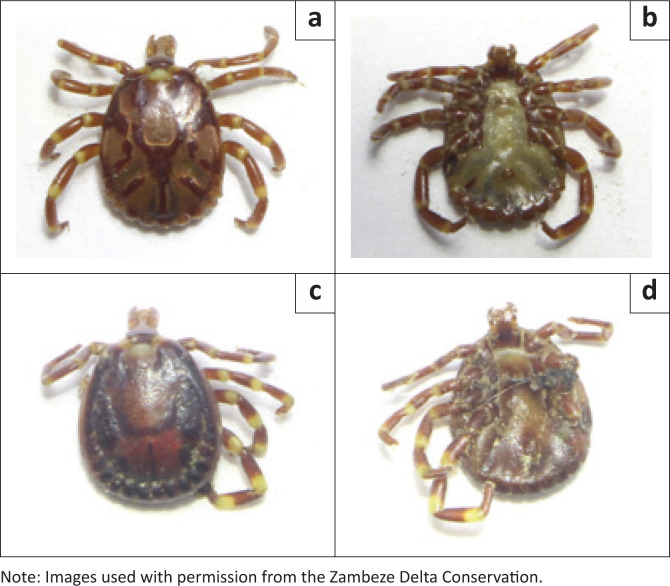
Dorsal and ventral views of two ticks collected during case 2, with (a and b) indicating *A. eburneum* and (c and d) indicating *A. variegatum*.

An adult female cheetah (~2.5 years) originating from Selati Game Reserve, South Africa, was released into the MCC in December 2023. Approximately 1 month post-release, the female suffered a deep wound to her lower back, presumably from a large predator encounter or hunting injury (Figure 1). Similarly, after sustaining the injury, the female developed an intense infestation of ticks in the periorbital, mandibular and genitoanal areas, which caused skin damage. The injury and tick infestation were detected during the weekly helicopter flight utilised for monitoring when the wet season conditions restrict vehicle access. Veterinary treatment was provided through manual tick removal, while antibiotics (Synulox RTU), vitamins (Catosal^TM^ B12) and fluids (NaCl 0.9%) were administered. The female died 2 weeks post-treatment, potentially because of septicaemia; however, the tick infestation had not returned. Fifteen ticks were collected during the treatment of this female and later identified as *Amblyomma variegatum* and *Amblyomma eburneum*, based on morphological characteristics with the assistance of taxonomic keys (Voltzit & Keirans 2003; Walker et al. 2003) and expert opinion (Figure 2).

### Ethical considerations

This study was conducted under a permit of research and data collection (ANAC RP #06/10/23) from the National Administration of Conservation Areas in Mozambique and ethical approval for research from the Institutional Animal Care and Use Committee, San Diego Zoo Wildlife Alliance (IACUC Protocol #23-010). The cheetahs were initially relocated under CITES import permits (MZ-00295/2022 and 23MZ000386).

## Discussion

In this study, we present evidence of high tick loads on reintroduced cheetahs, causing mild to significant damage to their periorbital regions. In both cases, the individuals were presumably immunocompromised after sustaining injuries during the peak hot-wet season. Given that several cases of natural and anthropogenic cheetah injuries have been observed in the MCC without the any sign of tick infestations, these two case studies suggest that tick infestations result from the interaction of three likely risk factors: (1) recent translocation exposing individuals to novel ectoparasites, (2) relatively severe injuries, and (3) the peak hot-wet season.

Both cheetahs were reintroduced into the MCC less than 4 months before developing tick infestations. It is typically during this period when cheetahs explore widely to familiarise themselves within their new environment and suffer high mortality rates because of anthropogenic pressures outside protected areas or encountering large predators (Weise et al. 2015). If these threats cause traumatic injury to cheetahs, it may disrupt immune system homeostasis (Stoecklein, Osuka & Lederer 2012). Evidence suggests that ticks may prefer unhealthy hosts, resulting in higher tick loads (Bunnell et al. 2011), as witnessed in our study. Chronic stress is an unavoidable component of animal translocations because of required phases of capture, temporary captivity, transport and release into a novel area (Dickens, Delehanty & Romero 2010). Although it is unclear how long the effects of chronic stress are experienced by animals post-release, it may have rendered these cheetahs more vulnerable to tick infestations as it also suppresses immune function (Dickens et al. 2010).

Recent translocation of these cheetahs could have exposed them to novel tick species given that distribution records for *A. eburneum* and *A. variegatum* show their occurrence within Mozambique, but not South Africa (Smit et al. 2024). It is plausible that these cheetahs had not yet acquired the immune responses required to fight off such tick infestations, as different tick species have diverse ranges of biologically active molecules that are differentially expressed in their saliva and modulate the host’s cutaneous and systemic immune defences (Wikel 2013). The hot-wet season could be another significant risk factor as a study of tick infestations in domestic ruminants found that tick abundance increases following rain, along with increasing average daily temperature, mean relative humidity and precipitation (Nasirian 2022). Literature on *A. eburneum* is scarce, but *A. variegatum* adults are known to be most abundant and actively feeding on hosts in the rainy season, which can lead to heavier infestations occurring in the warmer months (Petney, Horak & Rechav 1987; Walker et al. 2003). Nevertheless, potential for traumatic myiasis under such conditions remains poorly understood in wildlife because of the challenges of observing infested animals (Hall et al. 2016). While our study reports on a single incident, three incidents of myiasis were recently documented in cheetahs reintroduced to India, presumably aggravated by hot-wet conditions during the monsoon season (Jha 2023).

Admittedly, small sample size in this study restricts interpretation. However, given the similarities in both cases, we suggest that immunocompromised cheetahs are more prone to tick infestation during wetter periods in high rainfall areas. These cases highlight the significance of ectoparasites as a factor influencing cheetah survival post-reintroduction, particularly in tropical and subtropical ecosystems. Therefore, we recommend future cheetah translocations are prioritised during drier, cooler periods, an acaricide be applied before release, and intensive post-release monitoring be implemented to allow for timeous veterinary intervention. Ultimately, reintroduction and management of large carnivores remains complex, especially in modern-day Africa where anthropogenic pressures are mounting, although disseminating lessons learnt may contribute valuable insights for future conservation efforts and strategies of threatened species.
